# Mapping 50 Years of Sport Psychology–Performance Meta-Analyses: A PRISMA-ScR Scoping Review

**DOI:** 10.3390/sports13120420

**Published:** 2025-12-01

**Authors:** Marc Lochbaum, Andrew M. Lane

**Affiliations:** 1 Department of Kinesiology and Sport Management, Texas Tech University, Lubbock, TX 79409, USA; 2 Education Academy, Vytautas Magnus University, 44248 Kaunas, Lithuania; 3 Sport Physical Activity Research Centre (SPARC), University of Wolverhampton, Walsall WS13BD, UK; a.m.lane2@wlv.ac.uk

**Keywords:** quantitative reviews, competitive sports, athletics, performance enhancement

## Abstract

Sport psychology has generated an expansive body of literature on how psychological factors influence athletic performance, yet no review has systematically mapped this body of research. Guided by the PRISMA-ScR framework, this study charted published meta-analyses that investigated links between psychological constructs and sport performance. After conducting comprehensive searches, we identified 137 relevant papers, of which 73 met our inclusion criteria for athlete samples and performance outcomes. The included meta-analyses, published between 1988 and 2025, represented authors from at least 30 countries and covered more than 40 different constructs. Mental practice, confidence, achievement goals, anxiety, cohesion, mindfulness, and neurofeedback were the most frequently studied topics. Publication activity has accelerated rapidly since 2020, reflecting the maturation and diversification of evidence synthesis in sport psychology. Rather than aggregating effect sizes, this review maps methodological trends, recurring themes, and areas of limited coverage. The resulting catalog highlights where the evidence base is strongest, identifies emerging opportunities, and provides a foundation for future quantitative reviews to progress consensus development.

## 1. Introduction

Sport psychology has evolved into a globally recognized academic and applied discipline, with roots extending nearly 200 years. The beginning of sport psychology if we accept it as such with Carl Friedrich Koch’s 1830 publication [1], Calisthenics from the Viewpoint of Dietetics and Psychology [2]. Although the development of sport psychology has global roots [3], the field has been shaped primarily by Western scholarship [4,5,6,7,8,9,10,11,12,13,14,15,16,17]. Scholars have chronicled the evolution of the field and highlighted key figures, institutions, controversies, and trends over time. As such, the extent to which sport psychology constructs and findings apply across different cultural contexts remains in its infancy and requires more systematic exploration. Meta-analytic methods first entered the sport psychology literature in the 1980s, with landmark reviews by Feltz and Landers [18], Bond and Titus [19], and Mullen and Riordan [20]. To contextualize the scope of this review, we provide a brief review of meta-analyses entering the sport psychology literature and critically appraise our prior synthesis [21] to position the rationale for this scoping review.

### 1.1. The Rise of Meta-Analyses

The emergence of meta-analysis marked what seems to be a turning point for sport psychology as quantitative reviews offered a rigorous method for synthesizing findings across diverse studies and potentially signaling an expanding body of sport psychology literature. Although the statistical concept dates to Pearson’s [22] publication that is cited as foundational [23], it was not until 1983 that the first sport psychology-focused meta-analyses appeared, notably by Feltz and Landers [18] and by Bond and Titus [19]. A lesser-known contribution based on citations compared to the two 1983 meta-analyses, followed in 1988 by Mullen and Riordan [20], applying meta-analytic techniques to attribution theory. Also noteworthy is Meyers et al.’s [24] chapter titled Cognitive Behavioral Strategies in Athletic Performance Enhancement, which summarized 56 studies across 47 journals and remains a frequently cited resource. Whelan et al. [25] presented preliminary meta-analytic findings from this chapter at the American Psychological Association.

The buildup to published meta-analyses is, of course, grounded in single studies. Any one search is insufficient to capture the field’s scope. A Web of Science search using sport psychology as the keyword illustrates the scale of research growth: the first cataloged publication appeared in 1925 [26], followed by just 11 articles before 1970. That number grew to 288 in the 1970s, 880 in the 1980s, 1769 in the 1990s, 6939 in the 2000s, 14,561 in the 2010s, and 11,631 citations between 2020 and March 2025 alone. The volume reflects tremendous growth in the sport psychology published literature, particularly in western cultures. Feltz and Landers’ seminal meta-analysis on mental practice drew on 98 studies dating back to 1934. Bond and Titus conducted a cross-disciplinary meta-analysis on social facilitation, synthesizing 241 studies including Triplett’s [16] iconic work in cycling. Mullen and Riordan’s earliest citation was a Glyn C. Roberts conference presentation in 1975 [27]. These examples not only illustrate the historical reach of the sport psychology literature but also underscore the vital role meta-analysis plays in distilling insight from decades of findings.

Importantly, the rise of meta-analysis within sport psychology mirrors a broader scientific trend toward evidence synthesis and cumulative knowledge-building. Just as fields like medicine, education, and clinical psychology have relied on meta-analyses to inform best practice, sport psychology has adopted these methods to navigate its increasingly complex research base. This shift signals more than just methodological evolution. It reflects the growing credibility and relevance of sport psychology as both an academic discipline and an applied science. As will be detailed in the results, the post-2020 acceleration in meta-analytic publications speaks to global interest, rising methodological sophistication, and a commitment to generating practical, evidence-based guidance. In this way, meta-analysis is not just a tool for research consolidation, it is emblematic of the field’s maturity and its capacity to contribute meaningfully to both scientific discourse and societal outcomes in sport, health, and performance.

Building on the foundational studies described above, a systematic overview [21] summarized 30 sport psychology meta-analyses published between 1983 and 2021, spanning 16 psychological constructs. While the synthesis received constructive critique [28], it offered valuable insight: constructs hypothesized to enhance performance typically showed moderate effects, while those expected to impair performance demonstrated smaller, though statistically significant, associations. Since that review, the field has expanded rapidly, with a substantial number of new meta-analyses published across a broader range of psychological variables and performance outcomes, even by the same research group [29,30]. The volume and diversity of these newer reviews, particularly those emerging since 2020, have created both an opportunity and a need for a more comprehensive mapping of the evidence base [31].

A scoping review is therefore warranted to capture and organize this expanding body of research. The proliferation of meta-analyses examining constructs such as mindfulness, mental practice, emotional intelligence, confidence, and neurofeedback training has resulted in a complex and fragmented evidence landscape. While individual meta-analyses provide detailed quantitative insight into specific questions, the sheer number of overlapping and conceptually related syntheses now makes it difficult to discern the broader structure of knowledge in the field. A scoping review offers a systematic means of charting this terrain—identifying areas of concentration, redundancy, and inconsistency, while also highlighting underexplored topics. This broader synthesis provides a panoramic view of the evolution of sport psychology’s evidence base, supporting the development of more coherent theoretical frameworks and guiding future research priorities.

Further meta-analytic computation is not required at this stage. Many constructs have already been analyzed repeatedly, often drawing on similar primary studies, and additional aggregation would add little conceptual value. The strength of the current approach lies instead in synthesizing across existing quantitative reviews—collating and contextualizing their scope, methods, and findings to reveal patterns that are not visible within any single analysis. By mapping what has been studied, how it has been studied, and where evidence remains sparse, this scoping review provides an essential meta-level synthesis that integrates decades of quantitative research and identifies clear directions for future systematic reviews, empirical studies, and applied practice.

### 1.2. Purpose

The purpose of this review is to systematically map all published meta-analyses that examine psychological constructs in relation to sport performance. Extending our earlier synthesis of 30 meta-analyses [21], this study provides a substantially updated overview of the evidence base. Following the PRISMA-ScR framework [32], the review focuses on describing the breadth, methodological characteristics, and temporal trends of existing work rather than aggregating effect sizes or ranking constructs. By cataloging the scope of topics, research designs, and performance measures addressed across 73 meta-analyses, the review offers a resource to identify well-studied areas, highlight methodological gaps, and inform priorities for future quantitative synthesis and theory-driven investigation.

## 2. Materials and Methods

This review followed the Preferred Reporting Items for Systematic Reviews and Meta-Analyses extension for Scoping Reviews (PRISMA-ScR) guidelines [32], which are designed to map the scope and characteristics of research rather than statistically combine findings, as is done in traditional meta-analyses. This review was deliberately designed as a scoping review rather than a meta-analysis. In line with the PRISMA-ScR framework, the goal is to map and describe the breadth and characteristics of existing meta-analytic research in sport psychology, not to statistically aggregate or evaluate the quality of individual effect sizes. Because the purpose of a scoping review is to describe evidence coverage, we did not use quantitative synthesis, publication-bias testing, or heterogeneity statistics (e.g., I^2^); such analyses are outside the remit of PRISMA-ScR reviews. The completed PRISMA checklist is provided in Supplementary Table S1.

In terms of ethics, as this review synthesized previously published studies, it does not require new data collection or ethical approval. All included meta-analyses were based on primary studies that had already received ethical clearance from their respective institutional review boards. Rather than aggregating findings, the review uses a selective thematic summary of representative constructs to illustrate broader methodological trends while maintaining a comprehensive descriptive catalog of the literature.

The review was registered in PROSPERO (CRD420250653207).

### 2.1. Eligibility Criteria

The eligibility criteria for this review mirrored those used in Lochbaum et al. [21]. Meta-analyses were included if they were published in a peer-reviewed journal, examined a sport psychology construct, involved samples in which the majority of participants were described as athletes, and included meta-analytic data linking the psychological construct to sport performance. For the purposes of this review, athletes were defined as individuals formally engaged in organized sport or competitive training, from youth to elite levels. This operationalization distinguishes athletes from general exercisers or participants in non-competitive physical activity.

However, this review applied more stringent scrutiny to participant descriptions and the operationalization of sport performance outcomes. As a result, meta-analyses were excluded if they compared athletes to non-athletes as the primary focus, included when we could ascertain a number of included studies with participants described as children not engaged in youth sport, or assessed outcomes not directly tied to sport performance such as cognitive measures. Although the search was conducted in English, no formal English-language restriction was imposed.

### 2.2. Search Sources and Strategy

The lead author conducted the entire search and shared findings with the second author in email communications since June of 2023. The search process encompassed a number of iterations with most from published articles or databases. In summary, the origins started with a conference presentation [33] that led to Lochbaum and colleagues [21]. The search restarted with the lead author handsearching in June 2023, re-examining the 30 meta-analyses found in Lochbaum et al. [21], tapping into personal knowledge (i.e., lead author’s publications and notes from past work [34]), reading Holgado et al.’s [35] reference list. Then with a formal database search, the first author conducted searches in the first two weeks of March 2025 in EBSCOhost (APA PsychArticles, APA PsychInfo, ERIC, Medline, Psychology and Behavioral Science Collection, SPORTDiscus with Full Text), and WOS databases (Web of Science Core Collection 1900-present, Social Science Citation Index 1900-present, Emerging Sources Citation Index 2005-present, Medline). Since the formal search, Google Scholar alerts programed to find meta-analyses in sport psychology were reviewed while the manuscript was being written and revised.

**Figure 1:**
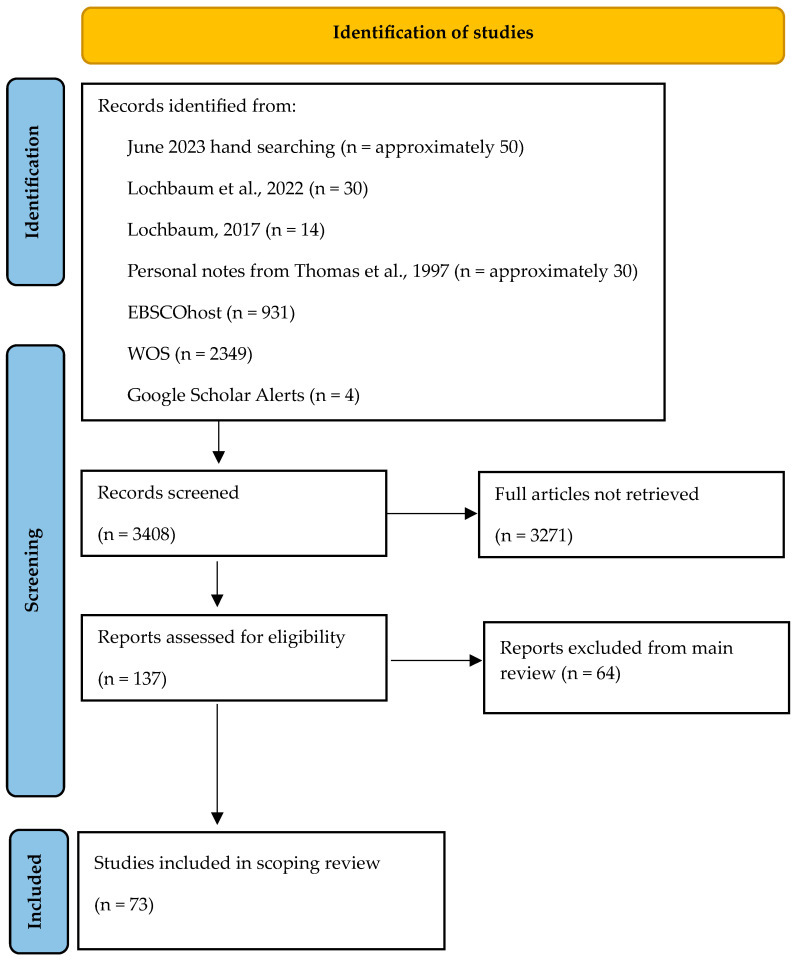
Search process flow diagram [21,33,34].

Within EBSCOhost (*n* = 931) and WOS (*n* = 2349), the following broad search was used with the goal of finding published sport psychology with performance data meta-analyses: (sport performance or competitive sport) AND (meta-analysis or meta analysis). Records were read from each search and notes compiled resulting in the 137 meta-analyses downloaded. Nearly all included meta-analyses were located in EBSCOhost and WOS in the March 2025 searches and within Lochbaum et al. [21]. Included articles found in Google Scholar alerts are also available now (searched to verify September 2025) in EBSCOhost and WOS. The search process is detailed in Figure 1. Details pertaining where each study was found are located in Table S2. The list of excluded meta-analyses is available from the lead author.

### 2.3. Data Items Retrieved

The following information was extracted from each meta-analysis: search result(s) (i.e., where found), APA 7th edition reference (from https://www.crossref.org/, EBSCOhost, WOS, or https://scholar.google.com/, 15 March 2025), country of all authors, topic, summary effect size statistic, manuscript stated purpose, number of studies, number of participants if provided, description of the sample, description of sport performance measure(s), most succinct author stated results, and author stated conclusion. Most of the stated results and conclusions came directly from the abstracts. When performance was one of many outcomes, the results and conclusions were located in the results section, summary tables, and discussion section.

## 3. Results

### 3.1. Summary Characteristics

A wide variety of topics were meta-analyzed with some meta-analyses examining more than one topic. Based on the publication year, the majority were published in the last decade (*n* = 49) compared to past decades: 1980s (*n* = 1), 1990s (*n* = 3), 2000s (*n* = 5), and 2010s (*n* = 15). From the titles and data retrieving, at least 40 topics are found within the 73 meta-analyses. The interest in conducting and publishing appears global. The countries of attributed to authors came from the following continents and countries: Africa: South Africa; Asia: China, Israel, Japan, Korea (Republic of Korea), Malaysia, Taiwan, Turkey; Europe: Austria. Belgium, Czech Republic, France, Germany, Greece, Ireland, Italy, The Netherlands, Poland, Slovenia, Spain, Sweden, Switzerland, UK; North America: Canada, Costa Rica, Grenada, USA; Oceania: Australia, New Zealand; and South America: Chile. Although this demonstrates wide international engagement, first author affiliations originated from Europe and North America were more evident, indicating that the evidence base remains weighted toward Western contexts.

The main sport psychology variables or topics are found in Table 1. Mental practice, achievement goals, anxiety, confidence, cohesion, mental fatigue, mindfulness, and POMS account for 32 the included meta-analyses. All of the included mental practice meta-analyses were published since 2020. Table 2 details the questions asked that is relationship between questions, effects of questions, and those reporting mean difference, ability to discriminate, influence of research questions and statistics were asked [20,29,36,37,38,39,40,41,42,43,44,45,46,47,48,49,50,51,52,53,54,55,56,57,58,59,60,61,62,63,64,65,66,67,68,69,70,71,72,73,74,75,76,77,78,79,80,81,82,83,84,85,86,87,88,89,90,91,92,93,94,95,96,97,98,99,100,101,102,103,104,105,106]. A few meta-analyses analyzed more than one question with one providing data on many different constructs. Most of the study samples were a mix of athlete levels (see Appendix A). As with the mix of samples, measures of sport performance varied greatly from one meta-analysis to another. No one metric can account for the variety and specifics (see Appendix A).

### 3.2. Individual Study Data

Table 3, Table 4 and Table 5 provide summary data for the 73 meta-analyses that met all inclusion criteria. For readers unfamiliar with typical effect size metrics in sport psychology, most meta-analyses report either correlation coefficients or standardized mean differences, such as Cohen’s d or Hedges’ g. While the interpretive thresholds for these metrics have been debated particularly the arbitrary nature of benchmarks in psychological research [107], commonly accepted guidelines in the social sciences classify correlations of 0.10 as small, 0.30 as medium, and 0.50 as large. For standardized mean differences, values of 0.20, 0.50, and 0.80 are typically used to represent small, medium, and large effects, respectively.

Table 3 summarizes the correlational findings and Table S3 contains more result specifics. Nearly all studies included 95% confidence intervals alongside the central effect size estimates. Of the reported findings, only one effect size, by Carron et al. [52], reached the conventional threshold for a large effect, though it was presented as a standard mean difference despite the analysis involving correlational data. Readers are strongly encouraged to consult the full meta-analyses for accurate interpretation, as many include bias-corrected estimates, true effect size calculations, and moderator analyses that offer deeper insight into the practical significance of findings. One recently published meta-analysis [95] addressed multiple psychological factors simultaneously. Ayranci and Aydin [95] provided an umbrella-style meta-analysis that included several psychological constructs such as motivation, confidence, self-efficacy, and emotional intelligence. We include this note here to acknowledge the overlap with other construct specific meta-analyses, though their broader scope and their search time frame 2014–2024 made the study difficult to classify as well as the initial reporting of the results using the correlation as the main metric but then reporting the single constructs (e.g., motivation, confidence) in Cohen’s d. Given the mean of the all the psychological constructs was reported in r, Ayranci and Aydin [95] is included in Table 3.

Table 4 summarizes findings from meta-analyses that examined the effects of interventions or psychological manipulations on sport performance outcomes with Table S4 containing more in-depth results. Compared to correlational studies, these reviews typically involved greater methodological complexity such as pre–post designs, active versus control comparisons, or intervention follow-ups which helps explain the wider range of observed effect sizes.

A few broad patterns emerge. Interventions such as biofeedback, mindfulness, process-goal setting, and psychological skills training often produced effects in the medium-to-large range, suggesting potential practical value for applied sport settings. In contrast, effects for constructs like ego-oriented goals, short-term breathing manipulations, and certain mental fatigue outcomes were negligible or inconsistent. These results highlight how both intervention design and the type of performance outcome measured could influence reported effectiveness.

Readers should note that these summaries cannot capture the full statistical detail. Many of the included reviews reported bias-corrected estimates, moderator analyses, or follow-up effects that provide richer context. Accordingly, we encourage readers to consult the original meta-analyses for deeper interpretation, particularly where intervention protocols or outcome definitions may affect applicability to specific sports or athlete populations.

Table 5 presents individual study details for 10 meta-analyses that addressed a range of research questions, including group differences, influence of variables, discriminative ability, and the magnitude of associations between sport psychology constructs and sport performance. Table S5 contains more detailed results. Of particular note, it was especially challenging to extract and condense the appropriate results for Weiß et al. [55], given the complexity of their analysis. Readers interested in those findings are encouraged to consult Table 5 of Weiß and colleagues’ original publication, which provides moderator analyses exploring the effects of different color types on performance outcomes.

As found in Table 5, although only ten meta-analyses are summarized, a few patterns emerge that highlight the diversity of research questions in sport psychology. Unlike the more commonly studied relationships (e.g., confidence–performance) or intervention effects (e.g., mental practice), these reviews explore constructs that do not fit neatly into those categories. Instead, they examine questions such as whether performance varies by goal orientation, emotional states, perceptual strategies, or attributional style. Further, the distribution of effect sizes is telling. Many findings fall into the “less than small” or “small” range, suggesting that certain popular constructs (e.g., performance-avoidance goals, mood states like anger and fatigue, or attributional explanations) may hold limited practical significance for predicting sport performance. By contrast, a handful of results rise to medium or large effects, notably in the case of the quiet eye literature, which consistently discriminates between successful and unsuccessful performances. Similarly, certain aspects of color and achievement goals show moderate associations, though these appear context dependent.

Overall, Table 5 underscores two points: first, that not all sport psychology constructs carry equal performance relevance, and second, that some areas (such as perceptual-attentional mechanisms like the quiet eye) demonstrate stronger and more consistent links with performance than many motivational or affective variables.

### 3.3. Interpretation and Potential Uses of the Results

Table 6, Table 7 and Table 8 focus on three frequently examined sport psychology topics: confidence, mental practice, and anxiety. Each has been the focus of multiple meta-analyses, enabling comparisons across time, methods, and inclusion criteria. For instance, self-confidence has been the focus of multiple meta-analyses. Four independent meta-analyses on confidence two published in 2003 [41,42] and two decades later [56,57] demonstrate striking consistency, despite differences in inclusion criteria and periods covered. As shown in Table 6, effect sizes range narrowly from 0.25 to 0.30. This stability suggests that while confidence is positively associated with performance, the effects are modest. As such, inflated claims that boosting confidence dramatically enhances sport performance should be treated with skepticism.

In contrast to confidence, the literature on mental practice presents more variation in both scope and interpretation. In contrast to the consistency found in confidence studies, meta-analyses on mental practice show wider variability in design, performance outcomes included, and conclusions (Table 7). Some reviews are broad in scope [69], while others focus narrowly on tennis [73]. The effects range from small to large depending on the outcome and whether mental practice was combined with physical training. Collectively, these findings reinforce the importance of context and raise questions about how mental practice is implemented and evaluated across sport domains.

Anxiety-performance relationships have long been of considerable interest in competitive sport. As found in Table 8, the first two meta-analyses were published in the 1990s [39,40] and the subsequent two meta-analyses published in 2003 [41,42]. Three of the meta-analyses suggest a negligible or unreliable relationship with performance. An exception is Jokela and Hanin [40], whose findings support the IZOF model. For the 2003 meta-analyses, the data in Table 8 represent cognitive anxiety and for the Woodman and Hardy meta-analysis the data reported in the table are from their ‘assuming 0 for missing correlations’ results. It is interesting that the last year for studies included in the four meta-analyses making up our cumulative knowledge of anxiety and sport performance was in 1998. Is there a need for an updated anxiety and sport performance meta-analysis or are we as researchers and practitioners to eliminate anxiety measurement from our research? Any quick search of the sport psychology and anxiety literature since 1998 clearly shows as a field we continue to research competitive anxiety and sport performance. Why would this research continue unless researched within the IZOF framework?

Beyond overall effects, many meta-analyses attempt to explain variability in findings through moderator analyses. However, these efforts vary in theoretical justification, statistical power, and reporting transparency. Suffice to say that moderators are a contentious topic. In Table 9, we present selected examples, as there are hundreds found in the 71 meta-analyses, that illustrate both the potential value and common limitations of moderator analysis in sport psychology research. The first example [75] shows differences by the performance measure classification, objective or subjective, for the mental toughness and performance relationship. With nearly twice the average mean effect, expectations of the value of mental toughness with objective performance measures should be dampened. Toth and colleagues [72] demonstrated a moderate mental practice and performance relationship that could and would if accurate impact the expected or hoped mental practice benefits on performance. If one is hoping for distance or time improvements, those are at best small whereas performance categorized as “other” is medium in meaningfulness. The other two examples point to different categorical moderators, goal type and sex of sample. What we learn from Williamson and colleagues [63] is that there are vastly different effect size values across process, performance, and outcome goals. Last, sex of sample across decades of studies appears to moderate the confidence-performance relationship [42,56]. Though lacking theory or any explanation, this result seems worthy of future research or do practitioners simply tell female athletes their confidence does not matter to their performances?

## 4. Discussion

The aim of this PRISMA-ScR review was to map the full range of meta-analyses examining psychological constructs in relation to sport performance. Seventy-three eligible reviews were identified, reflecting the rapid expansion and increasing methodological sophistication in Sport Psychology. Collectively, these reviews span more than 40 constructs, diverse athlete populations, and multiple performance outcomes.

Three broad patterns emerged. First is that the field has diversified dramatically since 2020, producing a wide range of theoretical perspectives, constructs, and analytic approaches. This heterogeneity is a defining feature of a maturing discipline: it reveals the multiple ways in which researchers have sought to understand psychology–performance relationship. Second, only a minority of meta-analyses have isolated specific athlete populations (e.g., elite performers) or focused exclusively on objective performance outcomes, meaning that practical applicability can vary across reviews. Third, the level of methodological detail reported across studies such as quality appraisal, bias assessment, and operational definitions of performance differs considerably, offering insight into how reporting conventions are evolving within the field. Rather than viewing these patterns as shortcomings, we interpret them as evidence of the field’s developmental breadth and as indicators of where conceptual and methodological alignment could yield greater cumulative understanding. The following section outlines realistic priorities for achieving such integration.

The scale of the reviewed meta-analytic literature is impressive but also daunting. What became apparent from our review efforts was not just the breadth of meta-analytic work, but also the fragmented nature of the evidence base. Most of the meta-analyses adopt broad inclusion criteria, use inconsistent terminology, and focus on a wide variety of sport performance measures. There is limited coherence across studies in terms of how they define target populations (e.g., recreational vs. elite athletes), how they conceptualize performance (e.g., objective measures like time or score versus subjective self-reports), and how rigorously they report methodological variables such as study quality or bias. This heterogeneity in reporting makes synthesis difficult and impedes the field’s ability to generate cumulative insights that inform practice or policy.

Despite the growth in the number of meta-analyses, relatively few are designed with a clear focus on athletes as a distinct population or on performance outcomes that are directly relevant to sport. Instead, many reviews aggregate across heterogeneous samples, contexts, and constructs. While some include moderator analyses that attempt to isolate athlete-level effects or differentiate between objective and subjective performance measures, these are inconsistently applied and often underpowered. Attempting to extract such information across all meta-analyses quickly became impractical. Moving forward, we see several avenues for progress:

1. Population-Specific Meta-Analyses: Future reviews should aim to isolate athlete populations more carefully, ideally differentiating by competitive level, sport type, gender, age, or training history. Stratifying samples in this way would enhance the ecological validity of findings and better reflect the diversity within sport. Without such granularity, it becomes difficult to draw conclusions that are meaningfully applicable to specific athlete groups or contexts.
2. Clearer Operational Definitions of Performance: Many reviews suffer from vague or inconsistent definitions of what constitutes ‘performance.’ Greater attention should be given to clarifying whether performance is measured through objective (e.g., competition results, biometric data) or subjective (e.g., coach ratings, self-report) means. Standardizing definitions and explicitly stating the measurement type would allow for more valid comparisons across studies and more direct translation into applied settings.
3. More nuanced moderator-focused reviews: By ‘more nuanced’, we mean analyses that move beyond basic demographic splits (e.g., gender or age) to explore interaction effects between multiple contextual variables such as competition level, sport type, intervention duration, or delivery format. These nuanced moderator analyses could help determine which interventions work best, for whom, and under what circumstances and ultimately bring us closer to the goal of tailored, context-specific guidance for applied practice. Achieving this will require both more detailed primary study reporting and a meta-analytic culture that prioritizes thoughtful moderator planning over sheer volume of included studies.
4. Thematic Synthesis: Beyond aggregating studies focused on single interventions, the field would benefit from reviews that integrate evidence around broader psychological constructs such as self-regulation, motivation, or emotion regulation. These thematic syntheses could reveal underlying mechanisms across interventions and promote a more theory-driven understanding of sport psychology’s contribution to performance and well-being.
5. Improved Reporting Standards: The consistent use of evidence synthesis guidelines—such as PRISMA, AMSTAR, and GRADE—can help ensure transparency and reproducibility. In particular, reporting of moderators, risk of bias, and heterogeneity should become standard practice. Doing so would not only enhance interpretability and comparability across meta-analyses but also elevate the overall methodological rigor in the field.
6. Societal Value and Employability: The volume of research in sport psychology could be better leveraged to demonstrate societal value and impact. Much of the evidence generated by meta-analyses—on performance, motivation, well-being, or behavior change—has relevance well beyond elite sport. Sport psychology graduates are equipped with analytical thinking, communication skills, and an understanding of human performance under pressure. These capabilities are valuable in sectors ranging from education and health to business and the military. Communicating the evidence base clearly, embedding findings into teaching curricula, and linking research to professional training and continuing professional development (CPD) can enhance both employability and the visibility of sport psychology as a discipline that serves broader societal needs.

### 4.1. De-Limitations and Limitations

Despite the comprehensive nature of this scoping review, several limitations should be acknowledged. Some reflect the boundaries intentionally set by design, whereas others became apparent during the review process. There were planned delimitations. The review followed PRISMA-ScR guidance, which prioritizes breadth of coverage over statistical synthesis. Accordingly, we did not perform a second-order meta-analysis, aggregate effect sizes, or conduct formal quality appraisal. These choices were deliberate to preserve inclusivity and transparency across a large and methodologically diverse evidence base. The search strategy, while extensive and multi-database (EBSCOhost, Web of Science, and Google Scholar alerts), was conducted in English, which may have introduced a language bias and excluded non-indexed sources. The inclusion criteria also focused specifically on meta-analyses that linked defined sport-psychology constructs to performance outcomes; therefore, studies examining related but indirect variables (e.g., cognitive or physiological markers) were not captured.

Concerning unplanned limitations, during synthesis it became clear that several features of the existing literature limited comparability in ways that were not fully evident at the outset. Many meta-analyses lacked consistent reporting of sample characteristics, effect-size metrics, or performance definitions, making it difficult to align data across studies even descriptively. In addition, a small number of eligible reviews had incomplete or ambiguous methodological information that could not be verified from the published record. Finally, the pace of new publications in 2024–2025 meant that several meta-analyses appeared during the writing phase, and although we incorporated as many as possible, the evidence base continues to evolve rapidly. Collectively, these limitations highlight both the strengths and constraints of scoping review methodology. They underscore the need for more standardized reporting and for dynamic, updateable databases to maintain currency as the field expands.

### 4.2. Immediate Next Steps

The volume of existing reviews is now sufficient to inform theory and practice; the greater challenge lies in integrating and coordinating what is already known. For this reason, we deliberately conducted a scoping review rather than another meta-analysis; we intentionally set out with the aim to map the extent and characteristics of existing evidence, expose overlaps and inconsistencies, and identify opportunities for more strategic synthesis. The results of this review highlight the need for greater coordination in how evidence is generated and shared across sport psychology. Two immediate actions could strengthen the field’s cumulative progress.

The first is to suggest establishing collaborative, open-access databases of primary studies would enhance transparency, reduce duplication, and enable large-scale moderator and bias analyses. Shared datasets would allow consistent tagging of variables such as intervention type, participant characteristics, and performance outcomes, improving comparability across future meta-analyses. Such infrastructure would also create valuable opportunities for early-career researchers to engage with high-quality data and contribute to cumulative, theory-driven science. Second, developing a Delphi-based consensus process involving journal editors, professional associations, and researchers from different regions and disciplines could help set priorities for future evidence synthesis. A structured Delphi study could identify under-researched topics, clarify reporting standards, and promote consistent definitions of performance and athlete populations. This collaborative process would ensure that future reviews address both established and emerging areas, supporting a more integrated and inclusive research agenda, e.g., refs. [108,109,110]. In short, the priority for future work is therefore not producing more meta-analyses, but improving how findings are connected, compared, and communicated. Coordinated use of shared databases, consensus frameworks, and consistent reporting standards will deliver greater progress than the continued proliferation of isolated reviews.

## 5. Conclusions

In conclusion, this PRISMA-ScR scoping review mapped 73 meta-analyses examining psychological constructs in relation to sport performance, illustrating both the breadth and methodological diversity of the field. The rapid growth of meta-analytic research reflects the maturity and international scope of sport psychology, with contributions spanning multiple theoretical traditions, performance domains, and athlete populations. Rather than viewing this diversity as a limitation, it should be seen as evidence of a dynamic and expanding discipline that now stands ready for greater synthesis and coordination.

The next stage of progress lies not in producing more isolated meta-analyses, but in integrating and harmonizing the substantial body of evidence that already exists. Establishing shared reporting standards, open-access databases, and collaborative consensus processes, for example, Delphi initiatives would facilitate methodological consistency, reduce duplication, and enhance the cumulative value of future research. Professional and scientific organizations, including the International Society of Sport Psychology (ISSP), national sport psychology associations, and journal editorial boards, could play a leading role in supporting such Delphi-based collaborations. By endorsing structured consensus exercises, these bodies can help align definitions, prioritize research questions, and encourage sustainable, collective approaches to evidence synthesis. By focusing on conceptual alignment, transparency, and collaborative infrastructure, sport psychology can move from mapping diversity to building coherence, generating an evidence base that is both scientifically robust and practically applicable to performance and well-being across contexts.

## Figures and Tables

**Table 1:** Topics represented in the sport psychology and sport performance meta-analyses included in the main review.

*n*	Topics
5	Mental practice
4	Achievement goals, Anxiety, Confidence
3	Cohesion, Mental fatigue, Mindfulness, Neurofeedback training, Profile of Mood States (POMS)
2	Attentional focus, Color, Decision training, Motor cortex stimulation (tDCS), Music, Perfectionism, Psychological interventions, Quiet eye, Self-efficacy, Self-serving attribution bias
1	Autonomy support, Biofeedback, Brain rhythms, Breathing techniques, Burnout, Coach education, Coping, Emotional intelligence, Flow, Goal setting, Home-field advantage, Interventions, Mental preparation techniques, Mental toughness, Perceptual anticipation, Personality, Pre-performance routines, Pressure training, Psychological Factors, Psychological Interventions, Self-talk, Stress regulation, Self-control

**Table 2:** Study reference, year published, main topic, and stated purpose for meta-analyses meeting all inclusion criteria (*n* = 73) listed in alphabetical order by topic.

Study	Year	Topic	Purpose
Lochbaum & Sisneros [29]	2024	Achievement goals	Relationship between situational and dispositional achievement goals and sport performance
Van Yperen et al. [36]	2014	Achievement goals	Relationships between achievement goals (MAp, PAp, MAv, PAv) and performance
Lochbaum & Gottardy [37]	2015	Achievement goals	To summarize the approach-avoidance achievement goal and performance in the sport psychology literature.
Ivarsson et al. [38]	2020	Achievement goals	Effect of psychological factors on future football (soccer) success
Kleine [39]	1990	Anxiety	Relationship between anxiety and sport performance.
Jokela & Hanin [40]	1999	Anxiety	Ability of the IZOF to discriminate between successful and less successful athletes
Craft et al. [41]	2003	Anxiety and confidence	Relationship between the CSAI-2 and sport performance
Woodman & Hardy [42]	2003	Anxiety and confidence	Relationship between anxiety and sport performance; and confidence and sport performance
Makaruk et al. [43]	2020	Attentional focus	Effect of attentional manipulations on jumping performance
Li et al. [44]	2022	Attentional focus	Effect of attentional focus on sprint performance
Mossman et al. [45]	2022	Autonomy support	Relationship between perceived coach autonomy support and performance
Lehrer et al. [46]	2021	Biofeedback	Effect of heart rate-variability training on performance
Filho et al. [47]	2021	Brain rhythms	Effects of brain rhythms on self-paced sport performance
Laborde et al. [48]	2022	Breathing techniques	Effects of breathing techniques on physical sport performance
Olsson et al. [49]	2025	Burnout	Relationship between burnout and athlete performance
Li et al. [50]	2025	Coach education	Effects of coach education training programs on athlete performance
Filho et al. [51]	2014	Cohesion	Relationship between cohesion and sport performance
Carron et al. [52]	2002	Cohesion	Relationship between cohesion and sport performance; effects of team cohesion on sport performance
Castaño et al. [53]	2013	Cohesion	Relationship between cohesion and performance (sport being one domain)
Peperkoorn et al. [54]	2024	Color	The influence of the color red on combat sport match outcome
Weiß et al. [55]	2024	Color	The influence of color on human behavior in the context of sport
Lochbaum et al. [56]	2022	Confidence	Relationship between pre-event confidence and sport performance
Jekauc et al. [57]	2023	Confidence	Relationship between pre-competition confidence and sport performance
Nicholls et al. [58]	2016	Coping	Relationship between coping strategies and sport performance
Silva et al. [59]	2021	Decision training	Effects of training programs on decision-making
Conejero Suárez et al. [60]	2020	Decision training	Effects of decision-making programs and interventions on decision-making
Kopp & Jekauc [61]	2018	Emotional intelligence	Relationship between emotional intelligence and sports performance
Harris et al. [62]	2021	Flow	Relationship between flow states and performance
Williamson et al. [63]	2022	Goal setting	Effect of goal setting on task performance
Jamieson [64]	2010	Home-field advantage	Effect of home-field on game outcome
Brown & Fletcher [65]	2017	Interventions	Effect of psychological and psychosocial interventions on sport performance
Clemente et al. [66]	2021	Mental fatigue	Effect of mental fatigue on soccer performance
Sun et al. [67]	2024	Mental fatigue	Effects of mental fatigue interventions on sport-specific outcomes
Habay et al. [68]	2023	Mental fatigue	Effects of mental fatigue on endurance performance
Liu et al. [69]	2025	Mental practice	Effects of imagery practice on enhancing athletic performance
Lindsey et al. [70]	2023	Mental practice	Effect of mental imagery programs on developing sport-specific motor skills
Simonsmeier [71]	2020	Mental practice	Effect of imagery interventions on sport performance
Toth [72]	2020	Mental practice	Effect of mental practice on performance (expressed as r)
Deng et al. [73]	2024	Mental practice	Effects of motor imagery training on tennis serve and return performance
Garzón Mosquera & Vargas [72]	2020	Mental preparation techniques	Effects of imagery-hypnosis interventions on performance
Hsieh et al. [75]	2023	Mental toughness	Relationship between mental toughness and athletic performance
Si et al. [76]	2024	Mindfulness	Effect of mindfulness interventions on athletes’ performance
Bühlmayer et al. [77]	2017	Mindfulness	Effect of mindfulness practice/interventions on sport performance
Ptáček et al. [78]	2023	Mindfulness-acceptance-commitment	Effects of Mindfulness–Acceptance–Commitment approach on performance
Maudrich et al. [79]	2022	Motor cortex stimulation (tDCS)	Effect (acute) of a single anodal tDCS session on sport-specific motor performance changes
Shyamali Kaushalya et al. [80]	2022	Motor cortex stimulation (tDCS)	Effect (acute) of transcranial direct current stimulation on cycling and running performance
Kämpfe et al. [81]	2010	Music	Effect of background music on performance
Terry et al. [82]	2020	Music	Effect of music in exercise and sport
Xiang et al. [83]	2018	Neurofeedback training	Effect of neurofeedback training for sport performance
Skalski et al. [84]	2025	Neurofeedback training	Effects of real-time EEG NFB in elite athletes
Yu et al. [85]	2025	Neurofeedback training	Effect of EEG neurofeedback training for sport performance
Zhu et al. [86]	2024	Perceptual anticipation	Effects of perceptual-cognitive training on anticipation and decision-making skills in team sports
Hill et al. [87]	2018	Perfectionism	Relationship between multidimensional perfectionism and sport performance
Kim et al. [88]	2025	Perfectionism	Relationship between multidimensional perfectionism and sport performance
Yang et al. [89]	2024	Personality	Relationship between Big 5 Model of personality and performance
Rowley et al. [90]	1995	Profile of Mood States-Mood	Ability of the POMS to discriminate between successful and less successful athletes
Beedie et al. [91]	2000	Profile of Mood States-Mood	Ability of the POMS to discriminate between successful and less successful athletes; Relationship between POMS and sport performance
Lochbaum et al. [92]	2021	Profile of Mood States-Mood	…tested whether the mental health model or iceberg profile is still characteristic of successful performances
Rupprectht et al. [93]	2021	Pre-performance routines	Effect of PPR interventions on sport performance
Low et al. [94]	2020	Pressure training	Effect of pressure training on performance
Ayranci & Aydin [95]	2025	Psychological factors	Relationships between psychological factors and sport performance
Kim et al. [96]	2021	Psychological interventions	Effect of psychological skills training for archery players
Reinebo et al. [97]	2023	Psychological Interventions	Effects of psychological interventions on sport performance
Barker et al. [98]	2020	Psychological interventions	Effects of psychological skills training and behavioral interventions on performance
Lebeau et al. [99]	2016	Quiet eye	Differences in quiet eye between experts and novices and within successful and unsuccessful performances
Sirnik et al. [100]	2020	Quiet eye/visual attention	Mean quiet eye duration and number of gaze fixations of successful shots
Hunte et al. [101]	2021	Self-control, prior exertion	Effects of self-control exertion on subsequent physical performance
Lochbaum et al. [102]	2023	Self-efficacy	Relationship between pre-event self-efficacy and sport performance
Moritz et al. [103]	2000	Self-efficacy	Relationship between self-efficacy and sport performance
Mullen & Riordan [20]	1988	Self-serving attribution bias	Magnitude of self-serving attribution biases for real-world sporting events
Allen et al. [104]	2020	Self-serving attribution bias	Magnitude of self-serving attribution biases for real-world athletic outcomes
Hatzigeorgiadis et al. [105]	2013	Self-talk	Effect of self-talk interventions on sport performance
Murdoch et al. [106]	2021	Stress regulation	Effect of stress regulation interventions on athletes

**Table 3:** Summary of ‘relationship between’ meta-analyses.

Meaningfulness	Topics
Less than small or non-significant	Achievement goals: ego climate and goal orientation [29]; mastery- and performance-avoidance goal [36]; Anxiety: CSAI-2 cognitive and somatic [41]; Perfectionism: concerns [87,88]; Personality: agreeableness, neuroticism, and openness to experience [89]
Small 0.10 to 0.29	Achievement goals: task climate and goal orientation [29], mastery- and performance-approach goal [36]; Autonomy support [45]; Cohesion: social [51,53] and task [53]; Confidence [41,42,56]; Emotional intelligence [61]; Perfectionism: strivings [87,88]; Personality: conscientiousness and extraversion [89]; Psychological factors [95]
Medium 0.30 to 0.49	Cohesion: overall [51], task [51]; Confidence [57]; Coping: mastery [58]; Flow [62]; Mental toughness [75]; Self-efficacy [102,103]; Mood: vigor [91]
Large ≥ to 0.50	Cohesion [52]
Small −0.10 to −0.29	Anxiety: Overall [39], cognitive [42]; Burnout [49]; Coping: internal regulation [58]; Mood: anger, depression, fatigue, and tension [91]
Medium −0.29 to −0.49	Coping: withdrawal [58]; Mood: confusion [91]
Large ≥ to −0.50	None reported in r

**Table 4:** Summary of effects of meta-analyses.

Meaningfulness	Topics
Less than small or non-significant	Achievement goals: ego orientation [38]; Breathing techniques: slow-paced, voluntary hyperventilation, breath-holding short-term [48]; Decision training technical execution [59]; Goal setting outcome goals [68]; Mental fatigue: running distance [66], shooting completion time [67]; Mental practice tennis speed, return accuracy [73];
Small 0.20 to 0.49	Achievement goals: task orientation and task-oriented coping [38]; Attentional focus: external > internal [43,44]; Mental practice [72]; Mental preparation techniques [74]; Mindfulness-acceptance-commitment [78]; Motor cortex stimulation (tDCS) [79,80]; Music [81,82]; Psychological (skills) interventions [97,98]
Medium 0.50 to 0.79	Brain rhythms [47]; Breathing techniques: slow-paced long-term [48]; Coach education [50]; Goal setting performance goals [68]; Home-field-advantage [64]; Interventions posttest [65]; Mental fatigue shooting [67]; Mental practice [68,69,70,71]; Neurofeedback training [83,84,85]; Perceptual anticipation training [86]; Pre-performance routines [93]; Pressure training [94]; Psychological interventions: mindfulness-acceptance and imagery [97]; Self-talk [105]; Stress regulation [106]
Large ≥ to 0.80	Biofeedback [46]; Decision training [59,60]; Goal setting process goals [68]; Interventions follow-up [65]; Mental practice tennis service accuracy [73]; Mindfulness [76,77]; Psychological (skills) interventions [97]
Small −0.20 to −0.49	Mental fatigue endurance performance [68]; Self-control prior exertion [101]
Medium −0.50 to −0.79	None
Large ≥ to −0.80	Mental fatigue shooting reaction time [67]

**Table 5:** Summary of meta-analyses with a variety of research questions not fitting into relationship between or effects of questions.

Meaningfulness	Topics
Less than small or non-significant	Achievement goals: performance- and mastery-avoidance [37]; Color win percentage only [100]; Mood: anger, fatigue, and tension [91,92]; anger, depression, and fatigue [91]
Small	Achievement goals: performance- and mastery-approach [37]; Anxiety, in zone [40]; Mood: vigor [91,92]; Mood overall [90]; Self-serving attribution bias: effort, difficulty, and luck [20], more personal responsibility for success than for failure [104]
Medium	Color [55]; Quiet eye within individual successful unsuccessful [99]; Achievement goals performance goal contrast [37]; Self-serving attribution bias: internal-external dimension [20,104]
Large	Quiet eye between successful unsuccessful [99]
Small and negative	Mood: confusion and depression [92]
Medium and negative	None reported
Large and negative	None reported

**Table 6:** Confidence meta-analyses.

Study	Studies	Samples	*n*	M	95% LL	95% UL	Years Covered
Lochbaum et al. [56]	41	49	3711	0.25	0.19	0.30	1986 to 2020
JeKauc et al. [57]	31	31	3107	0.30	0.21	0.37	2002 to 2021
Craft et al. [41]	29	55	2905	0.25	0.20	0.28	1984 to 1998
Woodman & Hardy [42]	36	36	2445	0.27	0.18	0.37	1981 to 1998

Abbreviations: *n* = number of total participants in the meta-analysis; M = mean effect size; LL = lower 95% confidence interval limit; UL = upper 95% confidence interval.

**Table 7:** Mental practice meta-analyses.

Study	Studies	M	LL	UL	Performance	Years Covered
Liu et al. [69]	86	0.50	0.34	0.67	Objective and subjective sport performance and physical performance measures	1990 to 2025
Deng et al. [73]	9	0.25	−0.02	0.53	Tennis serve speed	1998 to 2023
		0.98	0.66	1.30	Tennis serve accuracy	
		0.04	−0.40	0.48	Return accuracy	
Lindsey et al. [70]	10	0.61	0.31	0.90	Sport-specific motor skills	1985 to 2019
Simonsmeier [71]	48	0.41	0.27	0.55	Sport specific outcomes	
Toth [72]	37	0.20	0.12	0.28	Performance quantified according to distance (e.g., distance from the target), time (e.g., time to complete a task), or other (e.g., idiosyncratic scoring system).	1995 to 2018

Note: The Toth 2020 summary statistic is r. Abbreviations: M = mean effect size; LL = lower 95% confidence interval limit; UL = upper 95% confidence interval.

**Table 8:** Anxiety and performance meta-analyses.

Study	Studies	Samples	*n*	95% LL	M	95% UL	Years
Craft et al. [41]	29	55	2905	−0.03	0.01	0.04	1984 to 1998
Woodman & Hardy [42]	43	43	2445	−0.16	−0.10	0.03	1981 to 1998
Jokela & Hanin [40] (ES d)	19	41	3175	0.32	0.44	0.55	1978 to 1997
Kleine [39]	50	77	3589	−0.70	−0.19	0.59	1970 to 1988

Abbreviations: *n* = number of total participants in the meta-analysis; M = mean effect size; LL = lower 95% confidence interval limit; UL = upper 95% confidence interval.

**Table 9:** Study, topic, main result, and moderated result for selected meta-analyses.

Study	Topic	Main Result	Moderated Results
Hsieh et al. [75]	Mental toughness	r = 0.36	Objective performance r = 0.33
			Subjective performance r = 0.62
Toth et al. [72]	Mental practice	r = 0.13	Distance performance r = 0.06
			Time performance r = 0.11
			Other performance r = 0.31
Williamson et al. [63]	Goal setting	d = 0.47	Process goals d = 1.36
			Performance goals d = 0.44
			Outcome goals d = 0.09
Woodman & Hardy [42]	Confidence	r = 0.27	Male r = 0.29
			Female r = 0.04
Lochbaum et al. [56]		r = 0.25	Male r = 0.35
			Female r = 0.07

## Data Availability

All data are found within the manuscript.

## Supplementary Materials

The following supporting information can be downloaded at: https://www.mdpi.com/article/10.3390/sports13120420/s1, Table S1: Preferred Reporting Items for Systematic reviews and Meta-Analyses extension for Scoping Reviews (PRISMA-ScR) Checklist; Table S2: Included articles search source results; Table S3: Study, topic, and results expressed as r [95% confidence interval]; Table S4: Study, topic, and results for effects of meta-analyses; Table S5: Study, topic, and results for all other meta-analyses.

## Appendix A

**Table A1:** Included studies sample and sport performance measures.

Authors	Participants	Athletes	Measures
Allen et al. [102]	Athletes, club to elite	Mix	Real-world athletic outcomes
Ayranci & Aydin [95]	Athletes, elite to non-elite	Mix	Not stated but most likely mix of objective and subjective based on the single studies
Baker et al. [96]	Athletes, regional to elite	Mix	Variety of performance outcomes
Beedie et al. [90]	Athletes, non-elite to elite	Mix	Sport performance representing level of achievement and level of performance.
Brown & Fletcher [65]	Athletes, local, regional, national, or international	Mix	Variety of sport outcomes
Bühlmayer et al. [77]	Healthy sportive participants	Elite, recreational	Shooting and dart throwing
Carron et al. [52]	Athletes, youth to professional	Mix	Performance outcomes from interactive and coactive teams
Castaño et al. [53]	Athletes, reference suggest a mix	Mix	Vaguely described, win/loss ratio a stated example
Clemente et al. [66]	Athletes, youth, college, semi-professional	Mix	Tactical behavior in small-sided games
Craft et al. [41]	Athletes (predominantly) with college PE students	Mix	Variety of athletic performance outcomes
Deng et al. [73]	Athletes, novice to national level	Mix	Tennis serve and return measures
Filho et al. [47]	Experienced athletes and novices	Mix	Objective self-paced performance (e.g., golf putting, archery)
Filho et al. [51]	Athletes, recreational, interscholastic, collegiate, and professional	Mix	Objective and subjective sport measures
Habay et al. [68]	Athletes, university students to elite	Mix	Endurance performance measures including time trials and fitness tests
Harris et al. [62]	Athletes and gamers	Mix	Sport and computer gaming performance
Hatzigeorgiadis et al. [103]	Students, beginning athletes, experienced athletes	Mix	Variety of objective athletic measures
Hill et al. [87]	Athletes and some sport science students	Mix	Individual sport outcomes
Hsieh et al. [75]	Athletes, non-athletes, and recreational athletes	Mix	Subjective, objective athletic performance
Hunte et al. [99]	Athletes (recreational to trained for the motor results)	Mix	Objective sport performance measures (e.g., basketball freethrows) and one graded exercise test
Ivarsson et al. [38]	Athletes, adolescents	Youth, adolescents	Progression (e.g., higher level team), football statistics (e.g., goals, assists)
Jamieson [64]	Athletes, professionals from a variety of sports	Professional, collegiate	Game outcome
Jekauc et al. [57]	Athletes, youth to adult	Mix	Variety of objective, subjective performance measures
Jokela & Hanin [40]	Athletes, non-elite to elite	Mix	Criterion referenced, self-referenced, or subjectively rated sport performance
Kämpfe et al. [81]	Athletes, appears recreational and volunteer college students	Mix	Performance measures across a few sports and physical measures
Kim et al. [94]	Athletes, middle school to adults	Mix	Archery performance?
Kim et al. [88]	Athletes, variety of levels does not appear to include elite	Mix	Mix of objective measures, race time most frequent, only one non-sport (Yo-Yo test)
Kleine [39]	Athletes predominantly with general students in PE	Mix	Performance in many sports operationalized in multiple ways
Kopp & Jekauc [61]	Athletes, amateur to elite	Mix	Objective and subjective sport measures and physical parameters (e.g., maximal voluntary contraction)
Laborde et al. [48]	Athletes and active exercises	Mix	Objective sport specific and physical performance measures
Lebeau et al. [97]	Athletes, experts and non-experts	Mix	Self- and externally paced successful and unsuccessful sport performances
Lehrer et al. [46]	Athletes, difficult to find level	Mix, most likely	Athletic/artistic/performance
Li et al. [44]	Healthy > 18 years of age, low and high skill sprinters	Mix	Track sprinting
Li et al. [50]	Athletes, grades 4 to 12	Youth, adolescent	Objective psychomotor and game performance
Lindsey et al. [70]	Healthy volunteers ranging from novices to trained athletes	Mix	Sport-specific motor skills
Liu et al. [69]	Athletes of all ages no health requirement	Mix	Objective and subjective sport performance and physical performance measures
Lochbaum & Gottardy [37]	Athletes and non-athletes	Mix	Variety of objective and subjective sport measures including the PACER
Lochbaum & Sisneros [29]	Athletes, youth to elite	Mix	Variety of objective, subjective performance measures
Lochbaum et al. 2022 [56]	Athletes, youth to elite	Mix	Variety of objective, subjective performance measures
Lochbaum et al. [91]	Athletes, youth to elite	Mix	Variety of objective, subjective performance measures
Lochbaum et al. [100]	Athletes, youth to elite	Mix	Variety of objective, subjective performance measures
Low et al. [93]	Athletes, novice to elite	Mix (police officers not included)	Mostly self-paced skills in several sport contexts
Makaruk et al. [43]	Athletes, skilled and recreationally trained jumpers or untrained volunteers	Mix	Jumping performance
Maudrich et al. [79]	Healthy with at least 2 years of sport participation	Mix	Sports performance domains from many sports
Moritz et al. [101]	Athletes, non-athletes, and “other” category	Mix	Subjective and objective sport performance
Garzón Mosquera & Vargas [74]	Athletes, including professionals	Mix	Objective measures (e.g., golf performance, netball shooting)
Mossman et al. [45]	Participants in a sport or exercise setting distinct from physical education settings	Mix	Performance and achievement measures
Mullen & Riordan [13]	Athletes, reference titles suggests youths to college	Mix	Attributions in reference to real sport task
Murdoch et al. [104]	Athletes (defined as an individual who is behaviorally engaged in sport)	Mix	Objective and subjective sport measures
Nicholls et al. [58]	Vast majority late adolescent or young adult sport performers	Mix	Objective and subjective sport measures
Olsson et al. [49]	Athletes, club to international in track and field or swimming	Mix	Objective competition performance compared to personal best
Peperkoorn et al. [54]	Athletes, elite from boxing, taekwondo, and wrestling	Elite	Objective outcome, won/lose
Ptáček et al. [78]	Athletes, vague in description	Mix, most likely	Objective (sport and physical (e.g., strength) and subjective performance measures
Reinebo et al. [95]	Athletes competing at a regional or university level or higher.	Mix	Subjective, objective athletic performance
Rowley et al. [89]	Athletes, all levels	Mix	Sport performance such as personal best, ranking, selection for team, winning/losing, or subjective assessment.
Rupprectht et al. [92]	Athletes, 70% subelite and elite	Mix	Variety of performance outcomes
Shyamali Kaushalya et al. [80]	Healthy adults including trained athletes	Mix	Time to exhaustion, endurance time trial, and sprint performance
Si et al. [76]	Athletes, college to elite	College, elite	Objective, darts and appears a subjective questionnaire
Silva et al. [59]	Athletes, youth teams (regional to elite)	Mix	Objective and subject volleyball decision-making measures
Simonsmeier [71]	Athletes, novice to professional	Mix	Sport specific outcomes
Sirnik et al. [98]	Athletes, beginners to elite	Mix	Quiet eye duration for made basketball free-throw and jumpshots
Skalski et al. [84]	Athletes, defined as national, international-level	Elite	Objective with judo, archery, shooting most common; appears to include other performance variables (e.g., attention)
Conejero Suárez et al. [60]	Athletes, youth to 1st division	Mix	Objective and subject volleyball decision-making measures
Sun et al. [67]	Athletes, recreational to elite	Mix	Sport-specific outcomes from 6 sports
Terry et al. [82]	Athletes and exercisers	Mix	Objective performance in sports and physical activities
Toth [72]	Expert performers to novices	Mix	Performance quantified according to distance (e.g., distance from the target), time (e.g., time to complete a task), or other (e.g., idiosyncratic scoring system).
Van Yperen et al. [36]	Participants in sport domain	Mix, most likely	Performance on particular exercises, ranking in tournaments, outcomes of competitions, and assessments by coaches or trainers
Weiß et al. [55]	Participants in an actual sport/exercise setting, judging pictures/videos/real-life actions depicting a sport/exercise setting, imagining to be in a sport/exercise setting, or in virtual competition.	Mix	Variety of sport performance measures
Williamson et al. [63]	Athletes and non-athletes	Mix	Performance in darts, golf, basketball, volleyball, swimming, bowling, football, running, tennis, table-tennis, boxing
Woodman & Hardy [42]	Athletes, youth to at least semi-professional based on reference list	Mix	Variety of sport performance measures
Xiang et al. [83]	Athletes, recreational to elite	Mix	Performance in self paced sports including dance
Yang et al. [88]	Athletes, youth to elite	Mix	Objective and subjective sport measures
Yu et al. [85]	Athletes, including elite, and some novices	Mix	Objective with majority being precision movements (e.g., archery, shooting, dart throwing, golf putting)
Zhu et al. [86]	Athletes, recreational to elite	Mix	Task-specific response accuracy, response time, or on-court transfer RA/performance RT
