# Supplementary material for: Mapping 50 Years of Sport Psychology–Performance Meta-Analyses: A PRISMA-ScR Scoping Review
Source: Sports (Basel). 2025 Dec 1;13(12):420. doi: 10.3390/sports13120420 (PMC12737111; doi:10.3390/sports13120420)
Supplement: Supplementary file 1 [file sports-13-00420-s001.zip › sports-3956636-supplementary.pdf]

Table S1. Preferred Reporting Items for Systematic reviews and Meta-Analyses extension for Scoping Reviews (PRISMA-ScR) Checklist.

SECTION	ITEM	PRISMA-ScR CHECKLIST ITEM	Section, line # or area
		**TITLE**	
Title	1	Identify the report as a scoping review.	Title
		**ABSTRACT**	
Structured summary	2	Provide a structured summary that includes (as applicable): background, objectives, eligibility criteria, sources of evidence, charting methods, results, and conclusions that relate to the review questions and objectives.	See Abstract
		**INTRODUCTION**	
Rationale	3	Describe the rationale for the review in the context of what is already known. Explain why the review questions/objectives lend themselves to a scoping review approach.	Introduction + 1.1.
Objectives	4	Provide an explicit statement of the questions and objectives being addressed with reference to their key elements (e.g., population or participants, concepts, and context) or other relevant key elements used to conceptualize the review questions and/or objectives.	1.2. Purpose
		**METHODS**	
Protocol and registration	5	Indicate whether a review protocol exists; state if and where it can be accessed (e.g., a Web address); and if available, provide registration information, including the registration number.	2. Materials and Methods
Eligibility criteria	6	Specify characteristics of the sources of evidence used as eligibility criteria (e.g., years considered, language, and publication status) and provide a rationale.	Section 2.1. Eligibility criteria
Information sources	7	Describe all information sources in the search (e.g., databases with dates of coverage and contact with authors to identify additional sources), as well as the date the most recent search was executed.	2.2. Search sources and strategy
Search	8	Present the full electronic search strategy for at least 1 database, including any limits used, such that it could be repeated.	2.2. Search sources and strategy WOS EBSCOhost searches detailed
Selection of sources of evidence	9	State the process for selecting sources of evidence (i.e., screening and eligibility) included in the scoping review.	2.1. and 2.2.
Data charting process	10	Describe the methods of charting data from the included sources of evidence (e.g., calibrated forms or forms that have been tested by the team before their use, and whether data charting was done independently or in duplicate) and any processes for	2.3. Data items retrieved

		obtaining and confirming data from investigators.	
Data items	11	List and define all variables for which data were sought and any assumptions and simplifications made.	2.3. Data items retrieved
Critical appraisal of individual sources of evidence	12	If done, provide a rationale for conducting a critical appraisal of included sources of evidence; describe the methods used and how this information was used in any data synthesis (if appropriate).	Not appropriate
Synthesis of results	13	Describe the methods of handling and summarizing the data that were charted.	2.3.
**RESULTS**			
Selection of sources of evidence	14	Give numbers of sources of evidence screened, assessed for eligibility, and included in the review, with reasons for exclusions at each stage, ideally using a flow diagram.	2.2.
Characteristics of sources of evidence	15	For each source of evidence, present characteristics for which data were charted and provide the citations.	3.1. Summary characteristics, all references in reference section, and in tables
Critical appraisal within sources of evidence	16	If done, present data on critical appraisal of included sources of evidence (see item 12).	Not required
Results of individual sources of evidence	17	For each included source of evidence, present the relevant data that were charted that relate to the review questions and objectives.	Results section, see tables
Synthesis of results	18	Summarize and/or present the charting results as they relate to the review questions and objectives.	Results section, see tables
**DISCUSSION**			
Summary of evidence	19	Summarize the main results (including an overview of concepts, themes, and types of evidence available), link to the review questions and objectives, and consider the relevance to key groups.	Discussion section
Limitations	20	Discuss the limitations of the scoping review process.	4.2. De-limitations and Limitations
Conclusions	21	Provide a general interpretation of the results with respect to the review questions and objectives, as well as potential implications and/or next steps.	Section 5. Conclusions
**FUNDING**			
Funding	22	Describe sources of funding for the included sources of evidence, as well as sources of funding for the scoping review. Describe the role of the funders of the scoping review.	Stated after Author Contributions in the Funding declaration

Table S2. Included articles search source results (n = 73).

Authors	Year	Search
Allen et al. [102]	2020	EBSCOhost; WOS
Ayranci & Aydin [95]	2025	Google scholar alert August 30 2025
Baker et al. [98]	2020	WOS
Beedie et al. [91]	2000	Lochbaum et al. 2022; EBSCOhost; WOS
Brown & Fletcher [65]	2017	Lochbaum et al. 2022; EBSCOhost; WOS
Bühlmayer et al. [77]	2017	Lochbaum et al. 2022; WOS
Carron et al. [52]	2002	Lochbaum et al. 2022; EBSCOhost; WOS
Castaño et al. [53]	2013	Lochbaum et al. 2022; EBSCOhost; WOS
Clemente et al. [66]	2021	EBSCOhost; WOS
Craft et al. [41]	2003	Lochbaum et al. 2022; EBSCOhost; WOS
Deng et al. [73]	2024	WOS
Filho et al. [47]	2021	WOS
Filho et al. [51]	2014	Lochbaum et al. 2022
Haby et al. [68]	2023	WOS
Harris et al. [62]	2021	June 2023 hand searching; EBSCOhost; WOS
Hatzigeorgiadis et al. [105]	2013	Lochbaum et al. 2022; EBSCOhost; WOS
Hill et al. [87]	2018	Lochbaum et al. 2022; EBSCOhost; WOS
Hsieh et al. [75]	2023	June 2023 hand searching; EBSCOhost; WOS
Hunte et al. [101]	2021	June 2023 hand searching; EBSCOhost; WOS
Ivarsson et al. [38]	2020	Lochbaum et al. 2022; WOS
Jamieson [64]	2010	EBSCOhost; WOS
Jekauc et al. [57]	2023	June 2023 hand searching; EBSCOhost; WOS
Jokela & Hanin [40]	1999	Lochbaum et al. 2022; WOS
Kämpfe et al. [81]	2010	EBSCOhost; WOS
Kim et al. [88]	2021	EBSCOhost
Kim et al. [96]	2025	Google scholar alert July 2025
Kleine [39]	1990	Lochbaum et al. 2022; EBSCOhost
Kopp & Jekauc [61]	2018	Lochbaum et al. 2022; EBSCOhost; WOS
Laborde et al. [48]	2022	June 2023 hand searching; EBSCOhost; WOS
Lebeau et al. [99]	2016	Lochbaum et al. 2022; WOS
Lehrer et al. [46]	2021	EBSCOhost
Li et al. [44]	2022	June 2023 hand searching; WOS
Li et al. [50]	2025	WOS
Lindsey et al. [70]	2023	June 2023 hand searching; WOS
Liu et al. [69]	2025	Google scholar alert May 20 2025; EBSCOhost; WOS
Lochbaum & Gottardy [37]	2015	Lochbaum et al. 2022
Lochbaum & Sisneros [29]	2024	Author knowledge; EBSCOhost; WOS
Lochbaum et al. [56]	2022	Author knowledge; WOS
Lochbaum et al. [92]	2021	Lochbaum et al. 2022; WOS
Lochbaum et al. [102]	2023	Author knowledge; EBSCOhost; WOS
Low et al. [94]	2020	Lochbaum et al. 2022; WOS
Makaruk et al. [43]	2020	EBSCOhost; WOS
Maudrich et al. [79]	2022	Holgado et al. 2024 reference list; WOS

Moritz et al. [103]	2000	Lochbaum et al. 2022; EBSCOhost
Mosquera & Vargas [72]	2020	WOS
Mossman et al. [45]	2022	June 2023 hand searching
Mullen & Riordan [20]	1988	EBSCOhost
Murdoch et al. [106]	2021	June 2023 hand searching; WOS
Nicholls et al. [58]	2016	EBSCOhost; WOS
Olsson et al. [49]	2025	EBSCOhost
Peperkoorn et al. [54]	2024	EBSCOhost
Ptáček et al. [78]	2023	June 2023 hand searching; WOS
Reinebo et al. [97]	2023	Hand searching; EBSCOhost; WOS
Rowley et al. [90]	1995	Lochbaum et al. 2022; EBSCOhost
Rupprectht et al. [93]	2021	Hand searching; EBSCOhost; WOS
Shyamali Kaushalya et al. [80]	2022	Holgado et al. 2024 reference list; WOS
Si et al. [76]	2024	EBSCOhost
Silva et al. [59]	2021	WOS
Simonsmeier [71]	2020	Lochbaum et al. 2022
Sirnik et al. [100]	2022	WOS
Skalski et al. [84]	2025	Google scholar alert July 16 2025
Suárez et al. [60]	2020	WOS
Sun et al. [67]	2024	Hand searching; EBSCOhost; WOS
Terry et al. [82]	2020	Lochbaum et al. 2022; WOS
Toth [72]	2020	Lochbaum et al. 2022
Van Yperen et al. [36]	2014	EBSCOhost; WOS
Weiß et al. [55]	2024	Hand searching; WOS
Williamson et al. [63]	2022	June 2023 hand searching; WOS
Woodman & Hardy [42]	2003	Lochbaum et al. 2022; EBSCOhost; WOS
Xiang et al. [83]	2018	Lochbaum et al. 2022; EBSCOhost; WOS
Yang et al. [89]	2024	WOS
Yu et al. [85]	2025	EBSCOhost via searching for Skalski in EBSCOhost
Zhu et al. [86]	2024	WOS

Table S3. Study, topic, and results expressed as r [95% confidence interval].

Study	Topic	Result
Lochbaum & Sisneros [29]	Achievement goals	Task climate 0.20 [0.14, 0.25]; ego climate −0.00 [−0.48, 0.05]; task orientation 0.17 [0.12, 0.23]; ego orientation 0.09 [0.03, 0.16]
Van Yperen et al. [36]	Achievement goals	Mastery approach 0.17 [0.10, 0.23]; performance approach, 0.15 [0.08, 0.22]; mastery 0.02 [-0.06, 0.09]; performance avoidance -0.04 [-0.12, 0.05]
Kleine [39]	Anxiety	-0.19 [-0.75, 0.16]
Woodman & Hardy [42]	Anxiety and confidence	Cognitive anxiety -0.13 [-0.19, -0.04]; confidence 0.27 [0.20, 0.34]
Craft et al. [41]	Anxiety and confidence	CSAI-2 cognitive anxiety 0.01 [-0.03, 0.04]; somatic anxiety -0.03 [-0.08, 0.01]; confidence 0.25 [0.20, 0.28]
Mossman et al. [45]	Autonomy support	0.18 [0.13, 0.30]
Olsson et al. [49]	Burnout	-0.26 [-0.35, -0.17]
Castaño et al. [53]	Cohesion	Social 0.16 [0.04, 0.28]; task cohesion 0.12 [0.01, 0.23]
Filho et al. [51]	Cohesion	Overall 0.34 [0.26, 0.34]; task 0.45 [0.39, 0.46]; social 0.11 [0.03, 0.22]
Carron et al. [52]	Cohesion	Correlation studies reported with SMD (Standardized Mean Difference) effect size 0.69, SD = 0.91; experimental SMD = 0.40, SD = 0.46
Jekauc et al. 57]	Confidence	0.30 [0.21, 0.37]
Lochbaum et al. [56]	Confidence	0.25 [0.19, 0.30]
Nicholls et al. [58]	Coping	Mastery 0.30 [0.20, 0.39]; internal regulation -0.10 [-0.17, -0.03]; withdrawal -0.35 [-0.46, -0.25]
Kopp & Jekauc [61]	Emotional intelligence	0.16 [0.11, 0.22]
Harris et al. [62]	Flow	0.31 [0.24, 0.38]
Hsieh et al. [75]	Mental toughness	0.36 [0.24, 0.47]
Beedie et al. [91]	Mood	Anger -0.27; confusion -0.40; depression -0.34; fatigue -0.13; tension -0.25; vigor 0.47
Hill et al. [87]	Perfectionism	Strivings 0.23 [.11, .35]; concerns 0.06 [-.01, .14]
Kim et al. [88]	Perfectionism	Strivings 0.21 [0.15, 0.26]; concerns 0.03 [-0.02, 0.14]
Yang et al. [89]	Personality	Neuroticism -0.08 [-0.27, 0.10]; openness -0.00 [-0.06, 0.06]; agreeableness 0.03 [-0.07, 0.14]; conscientiousness 0.18 [0.08, 0.27]; extraversion 0.15 [0.05, 0.20]
Ayranci & Aydin [95]	Psychological factors (search 2014-2024)	All psychological factors 0.25; Specifics reported in Cohen’s d
Lochbaum et al. [102]	Self-efficacy	0.31 [0.22, 0.40]
Moritz et al. [103]	Self-efficacy	0.38 [0.35, 0.41]

Table S3. Study, topic, and results for effects of meta-analyses.

Study	Topic	Results
Ivarsson et al. [38]	Achievement goals	There was a small, positive effect of task orientation on future football performance (Cohen's d = 0.28, 95% CI = 0.07, 0.50). There was a small, positive effect of task-oriented coping strategies on future football performance (Cohen's d = 0.20 95% CI = 0.11, 0.28). There was a trivial effect of ego orientation on future football performance (Cohen's d = 0.06, 95% CI = −0.03, 0.14).
Li et al. 2022 [44]	Attentional focus	In general, the EF condition outperformed the IF condition in sprint performance (g = 0.279, 95% CI [0.088, 0.470], p = 0.004).
Makaruk et al. [43]	Attentional focus	The findings of this analysis revealed that the EF condition displayed superior jumping performance relatively to the IF (SMD = 0.33, 95% CI 0.14 to 0.51, Z = 3.50, p < 0.001) and CON (SMD = 0.35, 95% CI 0.11 to 0.58, Z = 2.92, p < 0.001) conditions. The third meta-analysis did not find any significant differences between the internal focus and control conditions (SMD = 0.07, 95% CI -0.20 to 0.35, Z = 0.52, p > 0.05).
Lehrer et al. [46]	Biofeedback	The overall effect size favoring HRVB was -0.90 and -0.25 with outliers.
Filho et al. [47]	Brain rhythms	We found that (a) optimal performance is characterized by increased alpha (g = 0.62, p = 0.02) and theta (g = 0.74, p = 0.002) across the cortex; (b) during optimal performance the frontal lobe is more relaxed (higher alpha; g = 1.06, p = 0.18) and less busy (lower theta; g = 0.38, p = 0.08), in comparison to the other brain lobes; (c) for the same given task, experts' brains are more relaxed (higher alpha, g = 0.89, p = 0.34) and less busy (lower theta, g = 0.91, p = 0.54) than novices' brains.
Laborde et al. [48]	Breathing techniques	Results showed that SPB and BH were related to improved PSP, with large and small effect sizes for longer-term interventions, respectively. In short-term interventions, SPB, BH, and VH were unrelated to PSP.
Li et al. [50]	Coach education	For the CEPIs group, a moderate to large effect on performance such as psychomotor and game performance (g = 0.58, 95% CI [0.41, 0.74]; k = 28, t = 7.29, p < .001) were noted.
Conejero Suárez et al. [60]	Decision training	The main finding of the meta-analysis was that the use of decision-making training programs/interventions led to significant improvements in volleyball players' decision-making (Standardized mean difference = 0.94 with 95% confidence interval from 0.63 to 1.25), compared to normal active volleyball training.
Silva et al. [59]	Decision training	The results showed a significant beneficial effect of DM interventions on tactical behavior (ES = 1.12; p = 0.035; I^2 = 80.0%; Egger's test p = 0.066), whereas no significant effect of DM interventions on technical execution was found (ES = 0.74; p = 0.180; I^2 = 69.1%; Egger's test p = 0.873).

Williamson et al. [63]	Goal setting	Process goals had the largest effect on performance (d = 1.36) compared to performance goals (d = 0.44) and outcome goals (d = 0.09). No significant difference in performance was found between specific (d = 0.37) and non-specific goals (d = 0.72).
Jamieson [64]	Home-field advantage	A significant advantage for home teams was observed across all conditions (Mp = .604).
Brown & Fletcher [65]	Interventions	Psychological and psychosocial interventions were shown to enhance sport performance at post-test (Hedges' g = 0.57, 95 % CI = 0.22–0.92) and follow-up assessments (Hedges' g = 1.16, 95 % CI = 0.25–2.08).
Clemente et al. [66]	Mental fatigue	Results showed no significant effect of fatigue on total running distance (ES = 0.13; p = 0.307) and tactical behavior (ES = 0.56; p = 0.079).
Sun et al. [67]	Mental fatigue	Interventions … showed potential to mitigate the detrimental effects on sport-specific performance, particularly in shooting accuracy (ES = 0.591; p = 0.001), decision-making accuracy (ES = 0.553; p = 0.006), and reaction time (ES = -0.871; p < 0.001), however, not in completion time (ES = -0.302; p = 0.182).
Haby et al. [68]	Mental fatigue	The meta-analysis shows the effect of MF on endurance performance was on average slightly negative (g= -0.32, [95% CI -0.46; -0.18], p<0.001).
Simonsmeier [71]	Mental practice	Imagery was effective to enhance motor learning and performance (d = 0.468, 95% CI [0.303, 0.633)].
Lindsey et al. [70]	Mental practice	The overall analysis revealed a significant moderate effect (g = 0.754; 95% CI = 0.557, 0.951; p < .001) for MI on skill outcomes. Analysis indicated that MI has a significant effect on performance (g = 0.476, bias corrected). Further analysis revealed significant effects on performance outcomes for MI combined with physical practice and MI alone (g = 0.579 and 0.298, respectively).
Toth [72]	Mental practice	Mental (r = 0.205, 95% CI 0.12, 0.28, z = 4.659, p < 0.001) practice had a significant positive effect on performance.
Liu et al. [69]	Mental practice	The Bayesian meta-analysis showed a statistically significant effect (for imagery vs. no practice) [μ(SMD): 0.5, 95% CI: 0.34–0.67; HDI: 0.34–0.67; BF: 12.41], with moderate between study heterogeneity and low within-study heterogeneity [within I2: 34.06%, between I2: 65.94%].
Deng et al. [73]	Mental practice	The results indicated that motor imagery training improved service accuracy and technique but did not affect service speed or return accuracy in tennis players.
Garzón Mosquera & Vargas [74]	Mental preparation techniques	A positive ES was found for…for performance (ES = .339; n = 60; p <.05).
Si et al. [76]	Mindfulness	Mindfulness training improves…performance [SMD = 0.92, 95% CI (0.40, 1.43), p < 0.01]
Bühlmayer et al. [77]	Mindfulness	Moderate to large effects were observed for both psychological performance surrogates (SMD 0.72, 90% CI 0.46–0.98, p < 0.001,

		I2 = 14%) and performance outcomes in shooting and dart throwing (SMD 1.35, 90% CI 0.61–2.09, p = 0.003, I2 = 82%).
Ptáček et al. [78]	Mindfulness - acceptance - commitment	a small model-averaged effect size estimate for improving sport performance with moderate evidence (g = 0.37, 95% CI 0.00–0.79, BFes = 3.27)
Maudrich et al. [79]	Motor cortex stimulation (tDCS)	significant moderate standardized mean difference (SMD) favoring anodal tDCS to enhance sport-specific motor performance could be observed (SMD = 0.31, 95% CI 0.14, 0.49).
Shyamali Kaushalya et al. [80]	Motor cortex stimulation (tDCS)	An overall small effect was observed in favour of the anodal-tDCS condition (SMD = 0.22; 90% CI = 0.05, 0.39; P = 0.04).
Kämpfe et al. [81]	Music	background music had a small but positive impact on sports performance (r weighted = 0.15, N = 11 studies, n = 259, rmin = -0.02, rmax = 0.88).
Terry et al. [82]	Music	Music was associated with significant beneficial effects on … physical performance (g = 0.31, CI [0.25, 0.36]), perceived exertion (g = 0.22, CI [0.14, 0.30]), and oxygen consumption (g = 0.15, CI [0.02, 0.27]).
Xiang et al. [83]	Neurofeedback training	Significant effects of NFT were found for sport performance (SMD = 0.65, 95%CI = 0.28 to 1.03, p = .0006).
Skalski et al. [84]	Neurofeedback training	The meta-analysis revealed a moderate positive effect of NFT on sport motor tasks, with a Hedges's g of 0.78 with a 95% confidence interval (CI) of 0.49–1.07.
Yu et al. [85]	Neurofeedback training	The meta-analysis revealed a moderate positive effect of NFT on sport motor tasks, with a Hedges's g of 0.78 with a 95% confidence interval (CI) of 0.49–1.07.
Zhu et al. [86]	Perceptual anticipation	Perceptual-cognitive training positively influenced elite athletes' anticipation and decision-making. However, its transfer effect on real-game performance improvement (ES = 0.65) was inferior to laboratory performance improvement (ES = 1.51).
Rupprechtt et al. [93]	Pre-performance routines	Results showed a significant but small effect of PPRs on sport performance in pre–post designs (SMC = 0.31, 95% CI [0.18, 0.44]) and moderate-to-large effects in experimental designs, both under low-pressure (Hedges' g = 0.64, 95% CI [0.45, 0.83]) and pressurized conditions (Hedges' g = 0.70, 95% CI [0.24, 1.16]).
Low et al. [94]	Pressure training	The included studies had a large positive effect (g = 0.85, 95% CI [0.37, 1.34]). Sport only data SMD = 0.72 95% CI [0.45, 1.00].
Kim et al. [96]	Psychological interventions	The overall effect size of psychological skills training interventions for archery players was ES = 0.469 (Hedges' g), which was a small-to-moderate effect size.
Reinebo et al. [97]	Psychological Interventions	Significant results were found in three of the meta-analyses (I, II, and III). Psychological skills training (g = 0.83, 95% confidence interval 0.21–1.45), mindfulness- and acceptance-based approaches (g = 0.67, 95% confidence interval 0.01–1.32), and imagery (g = 0.75, 95% confidence interval 0.14–1.36) outperformed controls with moderate effects.

Baker et al. [98]	Psychological interventions	Unweighted mean delta = 0.34, SD = 0.48
Hunte et al. [101]	Self-control, prior exertion	while smaller negative effect sizes were found for studies that implemented …and motor skill ($g = -0.45$ [-0.71, -0.20], $Z = 3.47$, $p < 0.001$) tasks.
Hatzigeorgiadis et al. [105]	Self-talk	The analysis revealed a positive moderate effect size (ES = 0.48, 95% 0.38, 0.58).
Murdoch et al. [106]	Stress regulation	We found a positive and significant moderate overall effect of stress regulation interventions on performance outcomes (65 effects, $k = 21$, $N = 2022$, $g = 0.52$, 95% CI = 0.19, 0.84).

Table S5. Study, topic, and results for all other meta-analyses.

Study	Topic	Author results
Lochbaum & Gottardy [37]	Achievement Goals	The performance goal contrast had a moderate-to-large positive impact on performance (g = 0.74, Z = 6.52) followed by the small-to-moderate positive impact of the mastery (g = 0.38, Z = 9.38) and performance (g = 0.38, Z = 4.60) approach goal. Both of the avoidance goals (performance g = -0.15, Z = 1.91; mastery g = -0.11, Z = 1.77) had small negative effects on performance.
Jokela & Hanin [40]	Anxiety	The findings provide fairly good empirical support for the IZOF anxiety model, with an overall effect size (d) for the in-out of the zone notion of d = +0.44 (41 effect sizes, n = 3175).
Weiß et al. [55]	Color	The meta-analysis with random effects models indicated a medium significant effect ($(\hat{\theta}$= 0.56, 95%-CI [0.40, 0.72], p < .001) with substantial heterogeneity (I2 = 96.97%, t2 = 0.34, pQ < .001).
Peperkoorn et al. [54]	Color	we found 50.5% wins by red for the overall data, which was not a statistically significant bias. Analyses of close contests resulted in 51.5% red wins, also not significantly different from the null expectation of equal proportions.
Rowley et al. [90]	Mood	The overall effect size was calculated to be 0.15.
Beedie et al. [91]	Mood	The weighted mean of all studies in MA1 showed very small effects associated with level of achievement (M = 0.10, SD = 0.07).
Lochbaum et al. [92]	Mood	Based on Hill's commentary [28] and the accessibility of the data to the first author, the correlational data, a few samples, were removed. Anger g = -0.03 [-0.27, 0.21], Confusion g = -0.43 [-0.80, -0.06], Depression g = -0.37 [-0.70, -0.04], Fatigue g = -0.37 [-0.48, 0.21], Tension g = -0.20 [-0.54, 0.14], and Vigor g = 0.36 [0.11, 0.61]
Lebeau et al. [99]	Quiet eye	a moderate effect size (d = 0.58, 95% CI 0.34, 0.82) comparing QE periods for successful and unsuccessful performances within individuals. …revealed very large effect sizes for … performance (d = 0.84, 95% CI 0.61, 1.06).
Sirnik et al. [100]	Quiet eye / visual attention	Results show that for both jump shots with or without defense and free throws, a longer quiet eye durations and a lower number of gaze fixations are associated with better performance.
Allen et al. [104]	Self-serving attribution bias	sport performers have a tendency to attribute personal success to internal factors and personal failure to external factors (k = 40, standardized mean difference [SMD] = 0.62), a tendency to attribute team success to factors within the team and team failure to factors outside the team (k = 23, SMD = 0.63), and a tendency to claim more personal responsibility for team success and less personal responsibility for team failure (k = 4, SMD = 0.28).
Mullen & Riordan [20]	Self-serving attribution bias	The internal-external dimension and the ability dimension produced effects of moderate magnitude, whereas effort, difficulty and luck produced effects of small magnitude.
